# A Quantitative Study of Inhibitory Interneurons in Laminae I-III of the Mouse Spinal Dorsal Horn

**DOI:** 10.1371/journal.pone.0078309

**Published:** 2013-10-25

**Authors:** Erika Polgár, Camille Durrieux, David I. Hughes, Andrew J. Todd

**Affiliations:** Institute of Neuroscience and Psychology, College of Medical, Veterinary and Life Sciences, University of Glasgow, Glasgow, United Kingdom; Nathan Kline Institute and New York University School of Medicine, United States of America

## Abstract

Laminae I-III of the spinal dorsal horn contain many inhibitory interneurons that use GABA and/or glycine as a neurotransmitter. Distinct neurochemical populations can be recognised among these cells, and these populations are likely to have differing roles in inhibiting pain or itch. Quantitative studies in rat have shown that inhibitory interneurons account for 25-40% of all neurons in this region. The sst2A receptor is expressed by around half the inhibitory interneurons in laminae I-II, and is associated with particular neurochemically-defined populations.

Although much of the work on spinal pain mechanisms has been performed on rat, the mouse is now increasingly used as a model, due to the availability of genetically altered lines. However, quantitative information on the arrangement of interneurons is lacking in the mouse, and it is possible that there are significant species differences in neuronal organisation.

In this study, we show that as in the rat, nearly all neurons in laminae I-III that are enriched with glycine also contain GABA, which suggests that GABA-immunoreactivity can be used to identify inhibitory interneurons in this region. These cells account for 26% of the neurons in laminae I-II and 38% of those in lamina III. As in the rat, the sst_2A_ receptor is only expressed by inhibitory interneurons in laminae I-II, and is present on just over half (54%) of these cells. Antibody against the neurokinin 1 receptor was used to define lamina I, and we found that although the receptor was concentrated in this lamina, it was expressed by many fewer cells than in the rat. By estimating the total numbers of neurons in each of these laminae in the L4 segment of the mouse, we show that there are around half as many neurons in each lamina as are present in the corresponding segment of the rat.

## Introduction

Laminae I-III of the dorsal horn contain a large number of inhibitory interneurons that modulate sensory information before this is transmitted to the brain and to other regions of the spinal cord. It has been shown that in the rat these cells constitute ∼25–30% of the neurons in laminae I–II and 40% of those in lamina III. Immunocytochemical studies suggest that they are all GABAergic, with some also using glycine as a co-transmitter [1]–[3]. Most of these cells give rise to axons that form axodendritic and axosomatic synapses and generate postsynaptic inhibition, however, axons of some GABAergic neurons form axoaxonic synapses that presynaptically inhibit primary afferents [4], [5]. Several distinct anti-nociceptive and anti-pruritic roles have been suggested for the inhibitory interneurons [6]–[8], and it is likely that these are performed by different functional populations. There have therefore been many attempts to classify inhibitory interneurons in laminae I–III, based on developmental, morphological, electrophysiological or neurochemical criteria [2], [9]–[14].

The somatostatin receptor sst_2A_ is expressed at high levels in the superficial dorsal horn of the rat, and is present on 13-15% of neurons in laminae I-II [15]–[18]. We have provided both anatomical and electrophysiological evidence that the receptor is restricted to GABAergic cells [16], [19], and we therefore estimate that it is expressed by ∼50% of the inhibitory interneurons in this region [20]. Studies in the rat have indicated that four non-overlapping neurochemical populations can be identified among the inhibitory interneurons in these laminae, based on the expression of galanin, neuronal nitric oxide synthase (nNOS), neuropeptide Y (NPY) and parvalbumin [21]–[23]. Interestingly, the sst_2A_ receptor is present on virtually all of those that express galanin or nNOS, but few of those that contain NPY and none of the parvalbumin cells [20]. This indicates that the receptor is associated with specific types of inhibitory interneuron in the superficial dorsal horn.

Quantitative information concerning interneurons is important, as it allows the sizes of different populations to be determined. It also provides baseline data for studies of pathological states in which interneurons may have been lost [8], [24], [25]. Although we have quantitative data concerning interneuron numbers in the rat [1], [16], [20], [22] there is little corresponding information for the mouse, which is increasingly used in pain research, because of the availability of genetically altered lines. Significant differences between rat and mouse have been reported, for example in the pattern of expression of the TRPV1 receptor on nociceptive primary afferents [26], and it is therefore not safe to assume that the organisation of interneuron populations is the same in both species. The first aim of this study was to determine whether the proportions of neurons that are GABAergic in laminae I–III of the mouse are the same as those previously reported for the rat [1] and whether, as in the rat, cells with high levels of glycine are also GABA-immunoreactive [1], [3]. We also tested whether the expression pattern of sst_2A_ is similar in the two species. Since the distribution of neurokinin 1 receptor (NK1r) was used to define the border between laminae I and II [27]–[29], we determined the proportion of neurons that expressed NK1r, in order to allow comparison with data from the rat, in which nearly half of the neurons in lamina I are NK1r^+^ [16]. Finally, the stereological method that we used allowed us to estimate the total number of neurons in each of these laminae in the L4 segment of the mouse, and compare this with corresponding data for the rat.

## Materials and Methods

### Ethics statement

All experiments were approved by the Ethical Review Process Applications Panel of the University of Glasgow and were performed in accordance with the UK Animals (Scientific Procedures) Act 1986.

### Animals and tissue processing

Eleven adult male C57Bl/6 mice of either sex, weighing 18–30 g were deeply anaesthetised with pentobarbital (20 mg i.p.) and perfused through the left ventricle with fixative. This consisted of 1% formaldehyde/2.5% glutaraldehyde for 4 mice, 4% formaldehyde/0.2% glutaraldehyde for 3 mice and 4% formaldehyde for the remaining 4 animals. In all cases the formaldehyde was prepared from paraformaldehyde immediately before use. Following perfusion fixation, the L4 segments were removed from all animals and cut into 60 µm thick sections with a Vibratome. Transverse sections were used for all parts of the study.

### GABA and glycine immunoreactivity in laminae I-III

Sections from the 4 mice fixed with 1% formaldehyde/2.5% glutaraldehyde were processed for post-embedding immunocytochemistry [1]. The sections were treated for 30 mins with 1% sodium borohydride to reduce free aldehyde groups. They were then osmicated (1% OsO_4_ for 20 mins), dehydrated in acetone and flat-embedded in Durcupan resin. From each of the 4 mice, 2 Vibratome sections were mounted onto blocks of cured resin and trimmed such that the block face contained the whole of laminae I-III on both sides. A series of 16 semithin sections, each 0.5 µm thick, was cut and the 1^st^, 8^th^, 12^th^ and 16^th^ of these were stained with toluidene blue. Three of the sections were processed for immunocytochemical detection of GABA (6^th^ section) or glial fibrillary acidic protein (GFAP, 7^th^ and 9^th^ sections) [1], [30]. They were etched for 40 min in a saturated solution of sodium hydroxide in ethanol, treated in 1% sodium metaperiodate to remove osmium, and then incubated overnight at 4°C in primary antibody against GABA (1∶10,000) or GFAP (1∶1000). After rinsing, sections were incubated for 1 hour at room temperature in biotinylated donkey anti-rabbit IgG (Jackson Immunoresearch, West Grove, PA, USA; 1∶500) and then for 1 hour in ExtrAvidin peroxidase conjugate (Sigma, Poole, UK, catalogue number E-2886; 1∶1,000). They were then reacted with 0.05% 3,3′-diaminobenzidine in the presence of H_2_O_2_ for 15 minutes to reveal peroxidase activity, and the reaction product was intensified with 0.01% OsO_4_ for 5 minutes. For this part of the study antibodies were diluted in phosphate buffered saline containing 0.5 M sodium chloride. Details of all the primary antibodies used in this study are given in Table 1.

**Table 1:** Primary antibodies used in this study.

Antibody	Species	Dilution	Source, catalogue number
GABA	Rabbit	1∶10,000*	Sigma, A-2052
		1∶5000	
Glycine	Rabbit	1∶50,000*	DV Pow
GFAP	Rabbit	1∶1000	DAKO, Z0334
sst_2A_	Guinea pig	1∶2000	Gramsch, SS-870
NeuN	Mouse	1∶1000	Millipore, MAB377
NK1r	Rabbit	1∶10,000	Sigma, S8305
PKCγ	Rabbit	1∶1000	Santa Cruz sc211

* dilutions used for post-embedding method

The physical disector method [31]–[33] was used to obtain an unbiased sample of neurons from laminae I-III of the dorsal horn in each series of semithin sections, as described previously [1]. The reference (8^th^) section, which had been reacted with toluidene blue was initially examined through a 20× objective lens on a Nikon Optiphot II microscope equipped with a drawing tube. The outline of the dorsal horn and boundaries between laminae I, II and III (identified from the distribution of myelin) were drawn, and the position of the lamina III-IV border was determined from the Allen Brain Atlas (http://mousespinal.brain-map.org/). Neuronal nuclei, which could readily be distinguished from oligodendrocytes by their pale appearance, were drawn. In most cases it was possible to differentiate neurons from astrocytes, but if necessary this was confirmed by examining the adjacent sections, which had been immunostained for GFAP. The look-up (16^th^) section was then examined, and any neuronal nuclei that were still present were identified and excluded. The 12^th^ section (also stained with toluidene blue) was also examined to rule out the possibility that two different nuclei in the same position on reference and look-up sections were mistakenly counted as one [1]. In this way, all neurons for which the bottom surface of the nucleus appeared between the reference and look-up sections was included in the sample. These were then identified on the section reacted for GABA, and the presence or absence of immunostaining was recorded.

In order to test the hypothesis that glycinergic neurons in laminae I–III were also immunoreactive for GABA [1], [3], we reacted pairs of serial semithin sections with GABA and glycine antibodies, as described above. One pair from each mouse was photographed, and the resulting digital images were overlaid. Glycine-immunoreactive cells in laminae I–III were identified and then examined for GABA immunoreactivity.

### Sst_2A_ and NK1r expression by neurons in laminae I-III

Sections from the 4 mice fixed with 4% formaldehyde were incubated for 3 days at 4°C in a cocktail of primary antibodies against sst_2A_, NeuN and NK1r and these were revealed by overnight incubation at 4°C in species-specific fluorescent secondary antibodies raised in donkey and conjugated to Alexa 488 (Invitrogen, Paisley, UK) or to Rhodamine Red or DyLight 649 (Jackson Immunoresearch). Secondary antibodies were used at 1∶500 (Alexa 488 or DyLight 649 conjugates) or 1∶100 (Rhodamine Red conjugate). The sections were then incubated in DAPI, to reveal cell nuclei. All antibodies used in this part of the study were diluted in phosphate buffered saline containing 0.3 M sodium chloride and 0.3% Triton-X100.

Two sections from each mouse were scanned with a Zeiss LSM710 confocal microscope with Argon multi-line, 405 nm diode, 561 nm solid state and 633 nm HeNe lasers. Images stacks with a z-spacing of 1 µm were acquired through a 40× oil-immersion lens, (numerical aperture 1.3) with the pinhole set to 1 Airey unit. Overlapping fields were scanned in order to cover the whole of laminae I-III of the dorsal horn on one side. The image stacks were viewed with Neurolucida for Confocal software (MicroBrightField, Colchester, VT, USA) and analysed by using a modification [34] of the optical disector technique [31], [32], [35]–[37]. The outline of the grey matter was initially drawn, and the locations of the boundaries between laminae I, II and III were identified based on the distribution of immunoreactivity for NK1r (which stains a dense plexus of dendrites in lamina I) and sst_2A_ (which stains dendrites throughout laminae I and II, see below). The ventral border of lamina III was located as described above. The 5^th^ and 20^th^ optical sections in the z-series were designated as the reference and look-up sections. The NeuN and DAPI channels were initially viewed for all optical sections in the z-stack, and neuronal nuclei were identified by the presence of staining for NeuN and DAPI. The locations of all neuronal nuclei that were present on the reference section, or appeared in subsequent sections, but had disappeared by the look-up section, were plotted. The channels representing sst_2A_ and NK1r were then switched on, and the presence or absence of each type of immunostaining in the neurons that were included in the disector sample was recorded. Although the primary purpose of this part of the study was to identify sst_2A_-expressing cells, we also counted those that possessed the NK1r.

In the conventional disector method, the look-up and reference sections are placed close together, so that none of the structures being examined can fall between them. Because we carefully examined all intervening optical sections to identify nuclei that lay entirely between the reference and look-up sections, we were able to place these sections further apart, and therefore obtain a larger sample.

We also used these results to calculate the neuronal packing density per 100 µm length of spinal cord for laminae I, II and III. Since there was a variable degree of tissue shrinkage during the processing of the Vibratome sections, the actual depth of tissue analysed with the optical disector (15 µm) was equivalent to a greater tissue depth from the original piece of fixed spinal cord. To compensate for this, we multiplied the number of cells observed within the 15 µm disector by a correction factor (the measured thickness of the section in micrometres, obtained by scanning the full thickness with the confocal microscope, divided by 60 µm) [34]. To estimate the number of cells in each lamina within the entire L4 segment, we measured the lengths of L4 in the 4 mice that were used in this part of the analysis, as described previously [34].

To confirm the laminar location of sst_2A_ immunoreactivity, sections from each of the 4 mice were incubated overnight in antibodies against sst_2A_ and PKCγ, and then for 4 hours in secondary antibodies conjugated to Alexa 488 or Rhodamine Red. The PKCγ antibody stains a plexus of dendrites that delineates the inner half of lamina II in the rat [38]. These sections were then scanned, together with a transmitted light image obtained with dark-field illumination to reveal lamina II, which can be recognised by its dark appearance, resulting from the lack of myelin [39], [40].

### GABA and sst_2A_ immunoreactivity

The relationship between GABA and sst_2A_ immunostaining was examined in sections from mice fixed with 0.2% glutaraldehyde/4% formaldehyde, since glutaraldehyde fixation is required for optimal retention of GABA [30], [41]. Sections were treated for 30 mins with 1% sodium borohydride, and then incubated overnight in antibodies against GABA and sst_2A_, which were revealed with fluorescent secondary antibodies as described above. Four dorsal horns were scanned with the confocal microscope through the 40× oil-immersion lens from each of the 3 mice. Because penetration of GABA immunostaining into Vibratome sections is extremely limited [20], [22], [42], [43], a very short z-series that included the top surface of the section was scanned. Again, overlapping scans were performed to include the whole of laminae I-III for each dorsal horn.

Scans were analysed with Neurolucida for Confocal. The sst_2A_ channel was initially viewed and the outline of the dorsal horn, together with the lamina II-III border were drawn, as described above. The locations of all sst_2A_-immunoreactive cells that had somata appearing on the upper cut surface of the Vibratome section were plotted [20], [22], [42]. The GABA channel was then viewed, and the presence or absence of GABA immunoreactivity in each of the sst_2A_^+^ cells was noted.

### Antibody characterisation

The GABA and glycine antibodies have been shown not to cross-react with the other amino acid, or with aspartate, glutamate, taurine or β-alanine [44], [45]. The GFAP antibody, which was raised against GFAP extracted from cow spinal cord, detects a single protein band of the appropriate size [46] and does not label cells other than astrocytes [47]. The sst_2A_ antibody was raised against the C terminal 15 amino acids of the mouse receptor, conjugated to keyhole limpet haemocyanin (KLH), and staining is abolished by pre-incubation of this peptide (manufacturer's specification). The NeuN antibody was raised against cell nuclei obtained from mouse brain [48] and we have shown that it labels all neurons, but no glial cells, in rat spinal cord [16]. The NK1r antibody was raised against amino acids 393–407 of the rat receptor coupled to KLH, and staining is absent in the brains of mice lacking the NK1r [49]. The PKCγ antibody is directed against the C-terminus of the mouse protein. We have reported that it stains identical structures to a well-characterised guinea-pig antibody [22], and staining with the latter antibody is absent from the brains of PKCγ knock-out mice [50].

## Results

### GABA and glycine immunoreactivity in laminae I-III

**Figure 1:**
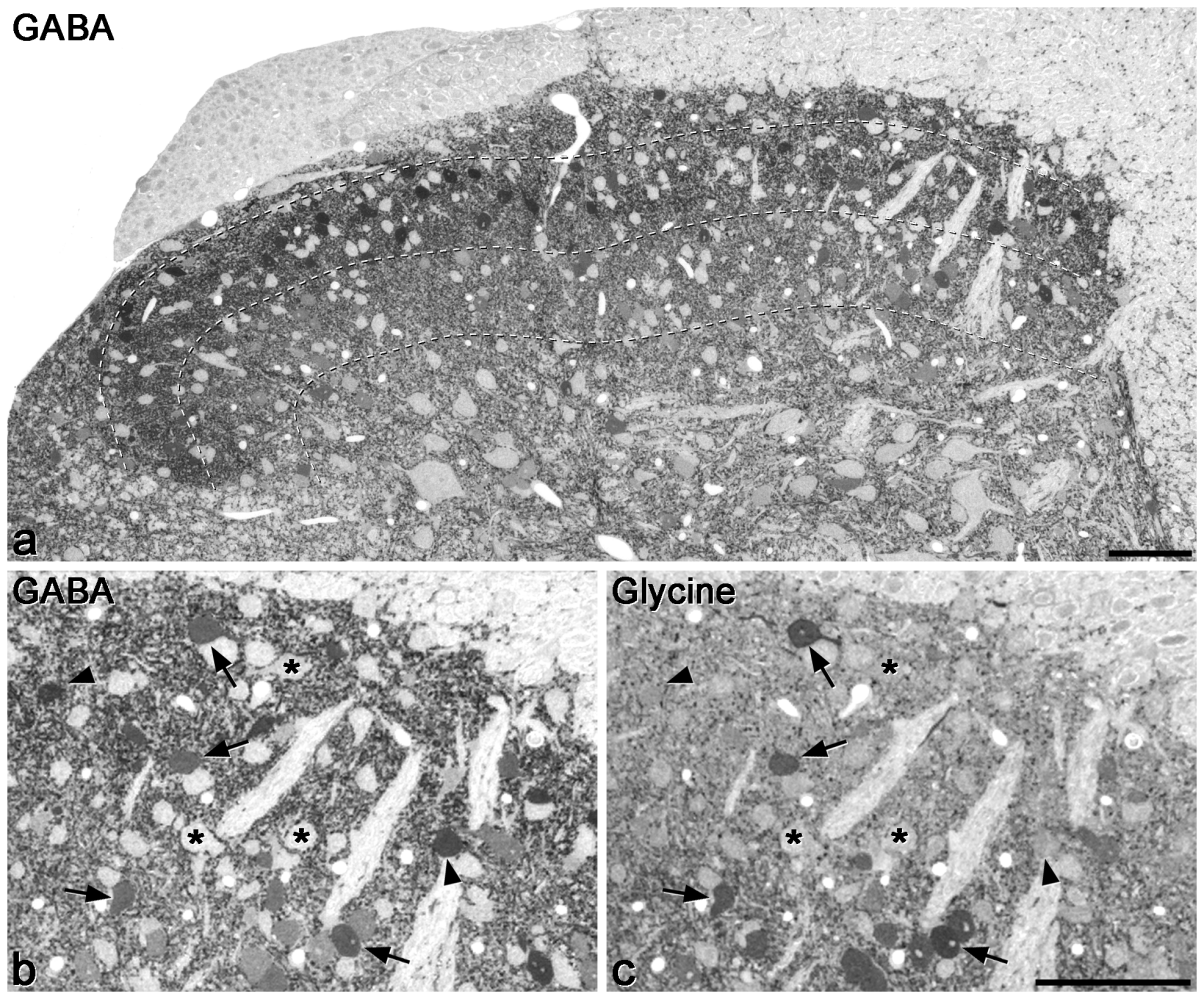
GABA and glycine immunoreactivity in a 0.5 µm thick transverse section of the mouse dorsal horn. **a** shows the whole mediolateral extent of the dorsal horn stained for GABA, while **b** and **c** show the medial part of the dorsal horn at higher magnification, in the same section and in a section reacted for glycine, respectively. Many GABA-immunoreactive cell bodies are scattered throughout the dorsal horn, and although these vary in intensity, they are clearly darker than the immunonegative cells, some of which are marked with asterisks in **b**. The dark staining in the neuropil represents GABAergic axons and dendrites. Dashed lines show the ventral borders of laminae I, II and III. **b** and **c** are serial semithin sections, so the same cells are visible. A few cells with relatively strong glycine immunoreactivity are visible (some marked with arrows) and these are also GABA-immunoreactive. In addition, there are cells that are GABA- but not glycine-immunoreactive (two marked with arrowheads). Scale bars  = 100 µm.

The distribution of GABA immunoreactivity was very similar to that reported in the rat [1], [3]. Numerous immunoreactive cell bodies were distributed throughout this region, and were also seen in lower numbers in deeper laminae (Fig. 1a). Non-immunoreactive cells could be readily distinguished by their uniform pale appearance. The neuropil showed a relatively high level of immunostaining, corresponding to dendrites of local interneurons and GABAergic axons. Quantitative analysis of the disector sample showed that in laminae I, II and III the proportions of neurons that were GABA-immunoreactive were 31%, 24% and 38%, respectively (Table 2).

**Table 2:** Results of the quantitative analysis of GABA immunoreactivity.

Lamina	Number of neurons sampled	Percent GABA-immunoreactive
I	46.5	30.5
	(33–64)	(24.2–32.8)
II	126.3	24.2
	(100–148)	(19.6–28.6)
III	116	37.6
	(110–121)	(32.2–42.6)

Mean values are shown with ranges in brackets (n = 4).

The pattern of glycine immunoreactivity was also very similar to that reported in the rat [1], [3]. In laminae I and II relatively few cells were immunoreactive, but immunoreactive cells were numerous in lamina III and in deeper parts of the grey matter (Fig. 1c). However, unlike the situation with GABA immunostaining, it was sometimes difficult to distinguish between weakly positive and negative cells. This could be because unlike GABA, which is only used as a neurotransmitter, glycine also has other roles, and the metabolic pool of glycine may vary between neurons. For this reason we did not attempt to quantify glycine-immunoreactive cells. However, we found that the great majority of cells in laminae I-III that had unequivocal glycine immunoreactivity were also GABA-immunoreactive (Fig 1b,c). Altogether, we examined 110 glycine-immunoreactive cells in laminae I-II (19–33/mouse, n = 4) and 203 cells in lamina III (33–62/mouse), and found that 109 (99%) of those in laminae I-II and 195 (96%) of those in lamina III were also GABA-immunoreactive.

### Sst_2A_ and NK1r immunoreactivity in laminae I-III

**Figure 2:**
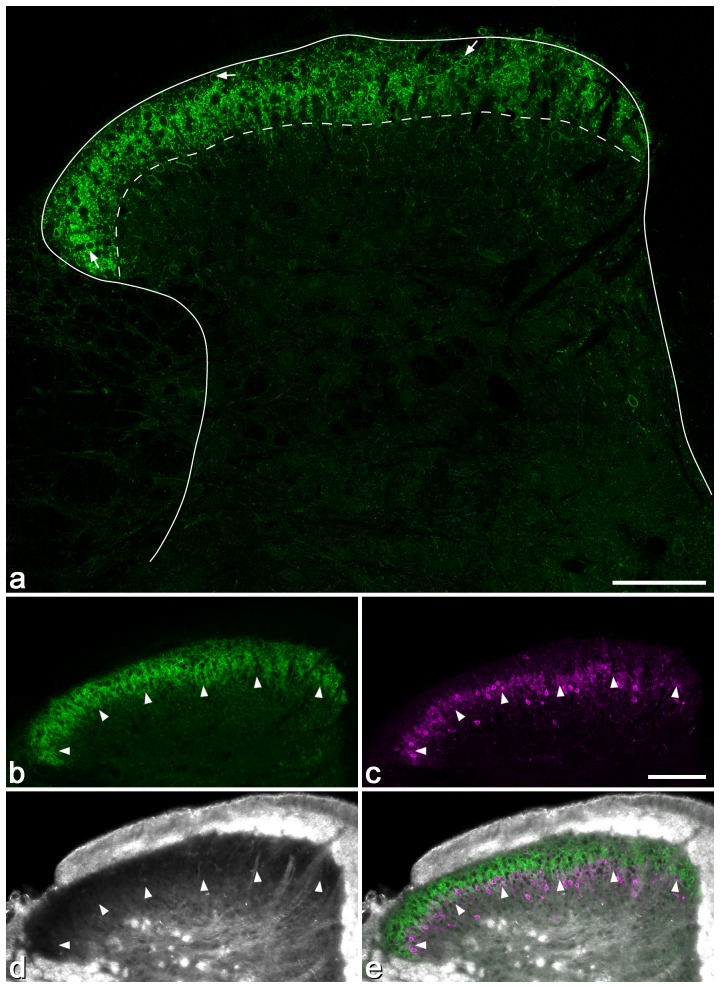
Distribution of sst_2A_ immunoreactivity in the mouse dorsal horn. **a**: There is a dense band of immunostaining in laminae I and II, with much lower levels elsewhere in the grey matter. Immunoreactive cells (some marked with arrows) are scattered throughout laminae I and II, and present at much lower density in deeper laminae. The solid line shows the grey-white matter boundary and the dashed line the border between laminae II and III. **b–f**: The relationship between sst_2A_ (green), PKCγ (magenta) and the appearance of the dorsal horn with dark-field illumination (DF). Note that although scattered PKCγ cells are present in lamina III, the ventral border of the PKCγ plexus (arrowheads) is at the same level as the ventral edge of sst_2A_ staining, and that this corresponds to the lamina II/III border as seen with dark-field microscopy. All confocal images are from single optical sections. Scale bar  = 100 µm.

**Figure 3:**
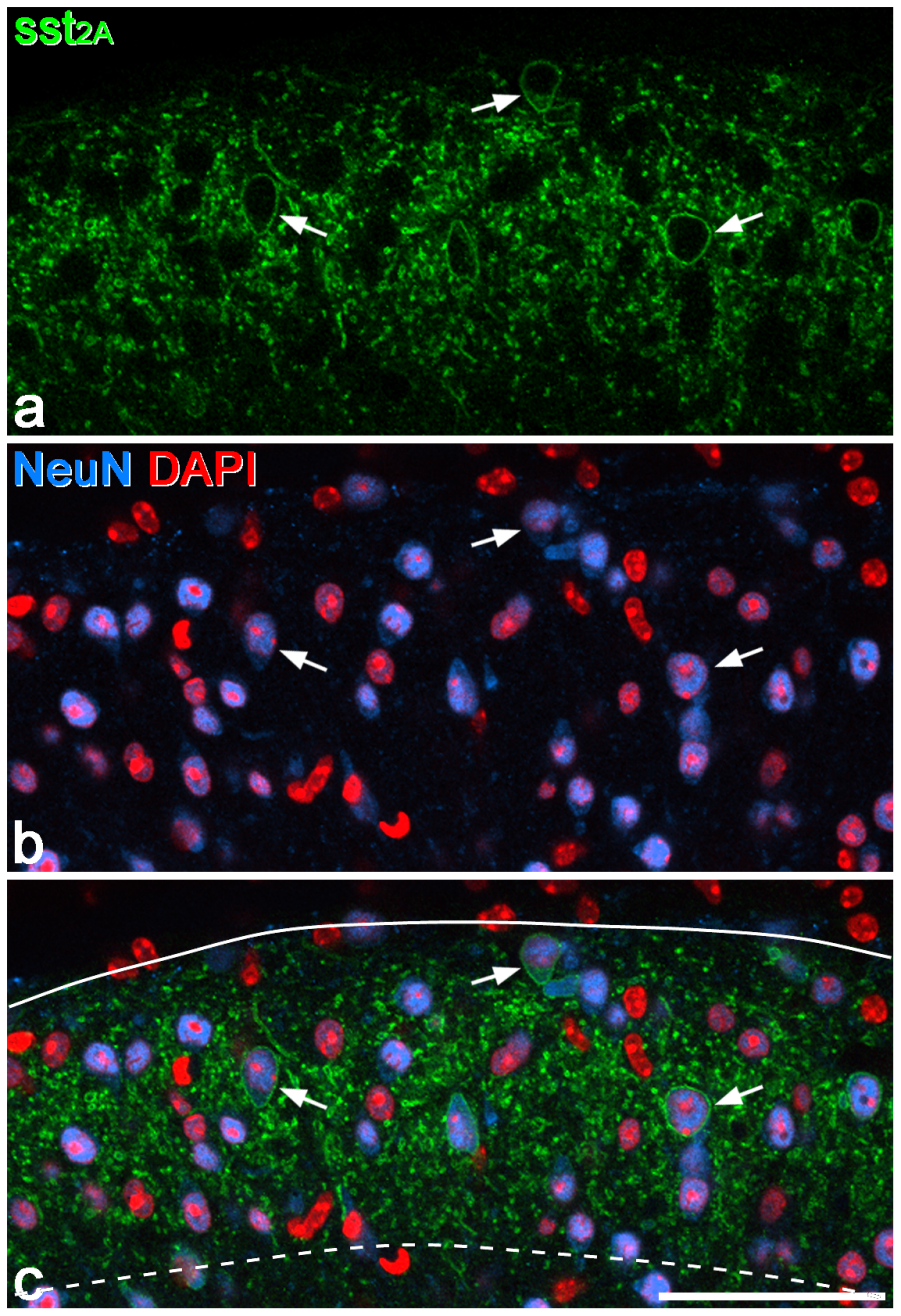
Confocal images showing sst_2A_-immunoreactive neurons in laminae I-II of the mouse dorsal horn in a transverse section used for quantitative analysis. **a**: Part of the section scanned to reveal sst_2A_ (green). Several immunoreactive cells are visible, and three of these are marked with arrows. **b:** The same field scanned to reveal NeuN (blue) and the nuclear stain DAPI (red). **c:** In the merged image, the dorsal border of the dorsal horn is shown with a solid line and the boundary between laminae II and III with a dashed line. In this image, neuronal nuclei appear magenta, while the nuclei of non-neuronal cells are red. Note that most neurons in laminae I-II are not sst_2A_-immunoreactive. The images were obtained from a single optical section. Scale bar  = 50 µm.

The distribution of staining for sst_2A_ was very similar to that reported in the rat [15], [16], with a dense band of immunoreactive cell bodies and dendrites occupying laminae I and II (Figs 2, 3). Staining was much weaker elsewhere in the dorsal horn, although a few immunoreactive cells were found in lamina III and occasionally in deeper laminae. Comparison of immunoreactivity for sst_2A_ and PKCγ showed that the ventral edge of the band of intense sst_2A_ staining corresponded to the ventral limit of the dense plexus of PKCγ, and this also matched the ventral border of lamina II, as defined with dark-field microscopy [38], [40]. Quantitative analysis with the optical disector method showed that sst_2A_-immunoreactive cells accounted for 17.3%, 12.8% and 6.5% of the neurons in laminae I, II and III, respectively (Table 3).

**Table 3:** Proportions of neurons in laminae I-III that were sst_2A_- or NK1r-immunoreactive and numbers of neurons in each lamina.

Lamina	Number of neurons sampled	% sst_2A_	% NK1r	Neuronal packing density (per 100 µm)	Estimated total number of neurons in L4
I	111	17.3	19.3	308	4466
	(88–139)	(13.6–19.8)	(14.4–25.5)	(255–346)	
II	334	12.8	4.8	940	13,630
	(314–370)	(11.9–14.3)	(2.4–8)	(875–1000)	
III	258	6.5	5.9	732	10,614
	(236–283)	(5–7.2)	(2.5–9.2)	(588–825)	

Mean values are shown with ranges in brackets (n = 4). The estimated total number of neurons in L4 is based on the average length of the segment (1.45 mm) in these 4 mice.

**Figure 4:**
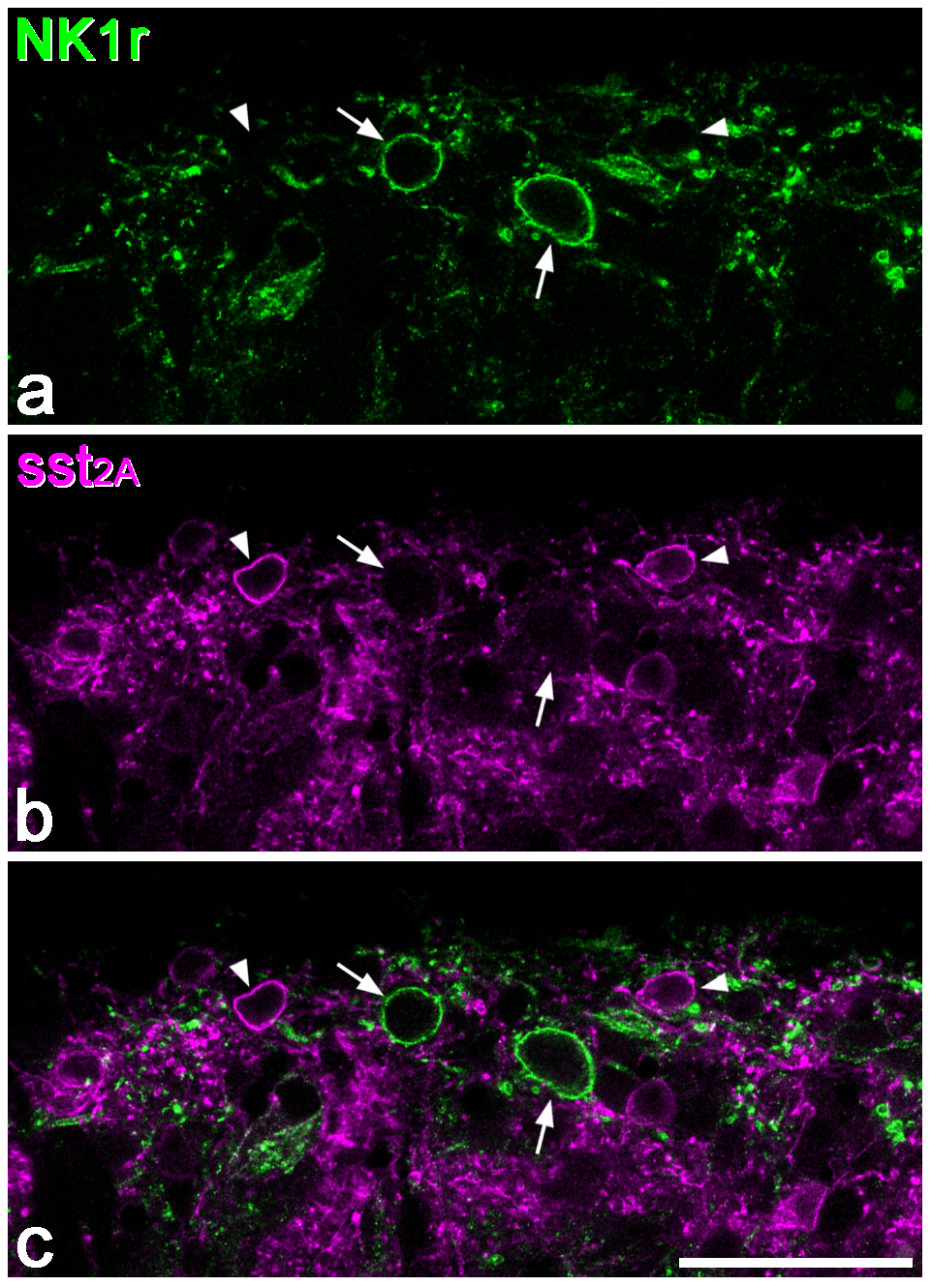
NK1r and sst2A in superficial dorsal horn. Confocal image showing a single optical section through part of laminae I and IIo, scanned to reveal NK1r (green) and sst_2A_ (magenta). **a**: Two NK1r-immunoreactive cell bodies (arrows) are visible in lamina I, surrounded by a plexus of labelled dendrites. **b**: Several sst_2A_-immunoreactive cells are visible in the same field (two shown with arrowheads). **c**: The merged image shows that none of these cells is labelled with both antibodies. Scale bar  = 50 µm.

As described previously in mouse [51]–[54] and rat [27]–[29], [55]–[57], NK1r-immunoreactivity was concentrated in lamina I, and present at lower levels throughout the deep dorsal horn (laminae III-VI), with very little in lamina II. NK1r-immunoreactivity was detected on 19.3%, 4.8% and 5.9% of neurons in laminae I, II and III, respectively (Table 3). Comparison of NK1r and sst_2A_ immunoreactivity in these sections showed that the NK1r antibody labelled a population of neurons that was almost entirely separate from those that were sst_2A_-immunoreactive (Fig 4). Altogether, 4 neurons that were double-labelled for both receptors were observed (2 in lamina I, 1 each in laminae II and III), and these represented 1.3% of the sst_2A_^+^ and 1.9% of the NK1r^+^ cells in this region.

### Neuronal packing density and numbers of neurons in each lamina

The packing densities of neurons in each lamina were determined from the numbers of neurons included in each disector (15 µm length) after correction for tissue shrinkage, and are shown in Table 3. The mean length of the L4 segment in the 4 mice used for this part of the study was 1.45 mm (range 1.39 – 1.54 mm), and from this we estimate that the total numbers of neurons in laminae I, II and III in this segment are 4466, 13,630 and 10,614, respectively (Table 3). These are between 50 and 60% of the sizes of the neuronal populations in these laminae in the rat L4 segment (7497, 27,465 and 21,928, respectively [34]).

### Expression of sst_2A_ by GABA-immunoreactive neurons in laminae I-III

**Figure 5:**
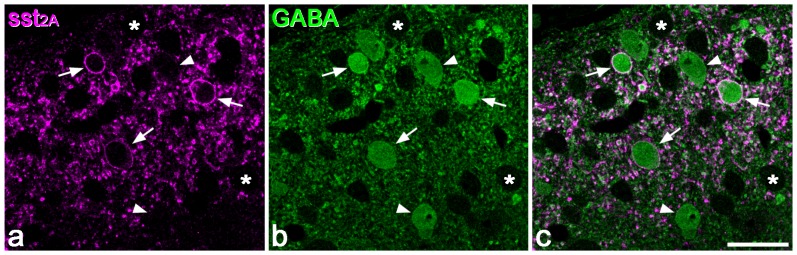
Restriction of sst_2A_-immunoreactivity to GABAergic neurons in the superficial dorsal horn. **a**: A confocal scan (single optical section) from the top surface of a Vibratome section that had been reacted to reveal sst_2A_ (magenta) and GABA (green). Three sst_2A_-immunoreactive cell bodies are marked with arrows, and there are many other labelled profiles, most of which are dendrites cut in cross section. **b**: The same field scanned to reveal GABA. Several GABA-immunoreactive cell bodies can be seen and some of these are indicated with arrows or arrowheads. Many cell bodies that are not GABA-immunoreactive are also present, and two are marked with asterisks. **c**: The merged image reveals that the sst_2A_-immunoreactive cells are all immunostained for GABA, but that several GABA^+^ cells do not possess sst_2A_ (two are indicated with arrowheads). Scale bar  = 20 µm.

The total number of sst_2A_-immunoreactive neurons in laminae I-II identified at the top surface of the sections from the mice fixed with 0.2% glutaraldehyde/4% formaldehyde was 177 (range 51–73, n = 3), and all of these were GABA-immunoreactive (Fig 5). Thirty-seven sst_2A_^+^ lamina III cells were seen in these sections (range 11–14), and most of these (mean 92%, range 75–100%) were also GABA-immunoreactive.

## Discussion

The main findings of this study are that 24–38% of the neurons in laminae I–III of mouse spinal cord are GABA-immunoreactive, with some also showing glycine immunoreactivity, and that (as in the rat) sst_2A_ is restricted to inhibitory interneurons in laminae I–II. However, although the laminar distribution of NK1r immunoreactivity is similar in both species, the number of immunoreactive cells is considerably lower in the mouse, with many fewer lamina I neurons showing NK1r-immunoreactivity.

### Inhibitory interneurons in laminae I–III

Inhibitory interneurons in the dorsal horn can use GABA and/or glycine as neurotransmitters [58]–[62], and we have previously reported that in the rat virtually all of the cells in laminae I–III that have high levels of glycine (and are therefore likely to use glycine as a transmitter) are also GABA-immunoreactive [1], [3]. Although we did not quantify glycine-immunoreactive cells in this study, we found that nearly all of the cells in laminae I–III that were clearly glycine-immunoreactive were also GABA-positive. This suggests that, as in the rat, GABA is present in the great majority of inhibitory interneurons in these laminae, with some of these (particularly in lamina III) using glycine as a co-transmitter.

Expression of the neuronal glycine transporter GlyT2 is required for glycinergic neurotransmission, and GlyT2 immunoreactivity is thought to be a reliable marker for glycinergic axons. However, the transporter is not present at detectable levels in neuronal cell bodies, and glycinergic neurons therefore cannot be identified by using immunocytochemistry with antibodies against GlyT2. Zeilhofer et al. [62] generated a bacterial artificial chromosome transgenic mouse in which enhanced green fluorescent protein (EGFP) is expressed under control of the GlyT2 promotor, and used this to examine the distribution of glycinergic neurons throughout the CNS. Although EGFP and glycine were generally co-localised in cell bodies in regions known to contain glycinergic neurons, there was a significant mismatch in the spinal dorsal horn. In particular, laminae I and II contained very few EGFP-positive cells, but several that were glycine-immunoreactive and EGFP-negative. Since the Allen Brain Atlas shows some cells with GlyT2 mRNA in laminae I-II of the mouse spinal cord, the discrepancy between glycine and EGFP reported by Zeilhofer et al. [62] may have resulted from lack of EGFP expression in glycinergic neurons in this region. However, it is also possible that some neurons that are not glycinergic have high levels of (metabolic) glycine, and caution is therefore needed when interpreting the results of glycine immunostaining.

If our assumption that nearly all glycinergic neurons in laminae I-III are also GABA-immunoreactive is correct, then based on the total numbers of neurons in each of these laminae (Table 3), and the percentages that are GABA-immunoreactive, we can estimate the proportions of neurons in this region that are inhibitory. According to this estimate, inhibitory interneurons account for 30.1% of all neurons in laminae I-III, and 25.8% of those in the superficial dorsal horn (laminae I-II). For comparison, the corresponding values for the rat are 33.9% for the whole of laminae I-III, and 29.9% for laminae I-II [1], [34].

This quantitative data for inhibitory interneurons will allow the proportion of these cells that belong to different sub-populations to be determined. For example, we have found that in the rat, GABAergic cells that contain nNOS, galanin or NPY account for over half of all the inhibitory interneurons in laminae I-II [22]. These neurochemical classes differ both in their responses to noxious stimulation [20] and also in their post-synaptic targets, with at least some of the nNOS- and NPY-containing cells being presynaptic to specific classes of projection neuron in laminae I and III, respectively [39], [63], [64]. It has recently been shown in the mouse that the parvalbumin-containing inhibitory neurons are involved in presynaptic inhibition of low-threshold mechanoreceptive primary afferents [65], providing further evidence that neurochemically-defined populations of inhibitory interneurons differ in function.

There are several mouse lines in which discrete populations of inhibitory interneurons can be identified by expression of EGFP [12], [65]–[68], and the results of this study will make it possible to determine directly the proportion of inhibitory interneurons that are included in each of these populations. They will also allow an accurate assessment of any neuronal loss that results from pathological conditions such as spinal cord or peripheral nerve injury [24], [25], [69], or from genetic alterations. For example, Ross et al. [8] have reported that exaggerated itching in mice lacking the transcription factor Bhlhb5 results from loss of inhibitory interneurons in laminae I-II of the dorsal horn. In contrast, Wang et al. [70] observed abnormal pain behaviours in mice in which the testicular orphan nuclear receptor TR4 had been deleted, and this was attributed to selective loss of excitatory interneurons from the superficial dorsal horn, which would presumably result in an abnormally high proportion of GABAergic neurons in this region.

### Sst_2A_ receptor expression

As in the rat, we found that all sst_2A_^+^ neurons in laminae I-II were GABA-immunoreactive [16]. From the relative numbers of neurons in these laminae, we estimate that 13.9% of all superficial dorsal horn neurons are sst_2A_-immunoreactive, and therefore that ∼54% of the inhibitory interneurons in this region express the receptor in the mouse. This is slightly higher than the corresponding estimate for the rat (48%) [20].

There has been controversy concerning the actions of somatostatin at the spinal level, with both pro- and anti-nociceptive effects being reported [71]-[76]. Anti-nociceptive effects have generally been seen with relatively high doses, which can cause significant neurotoxicity, possibly by reducing local blood flow [75]–[77]. In contrast, lower doses (up to 20 µg i.t. in rats) have resulted in signs that were interpreted as resulting from a pro-nociceptive action [71]–[73].

The Allen Brain Atlas shows high levels of mRNA for sst2, but not for the other somatostatin receptors, in the mouse superficial dorsal horn, and this is consistent with the distribution of sst_2A_ immunoreactivity reported in several studies in the rat [15]–[18], [78] and with the results of the present study. Sst_1_ receptors are also found in the superficial laminae, but these are presumably located on the central terminals of primary afferents (see below) [18]. Although one study has reported that the other sst_2_ splice variant (sst_2B_) is present throughout the grey matter in rat spinal cord [78], this does not match the distribution of sst_2_ mRNA in the mouse. This could conceivably reflect a species difference, but it is more likely that sst_2B_ is only be expressed at very low levels. Application of somatostatin to dorsal horn neurons results in hyperpolarisation [79]–[81], and we have shown that this effect is restricted to inhibitory interneurons in lamina II of the rat [19], consistent with our immunocytochemical findings for sst_2A_ in this species. These observations suggest that the direct action of somatostatin on dorsal horn neurons is mediated mainly or exclusively through sst_2A_ receptors. Somatostatin released from primary afferents or local excitatory interneurons [82]–[84] acting on these receptors should therefore result in disinhibition, and it is likely that this accounts for the pro-nociceptive effects that have been reported for intrathecal somatostatin [71]–[73]. However, both sst_1_ and sst_2A_ receptors are expressed by some primary afferents [17], [18], and a presynaptic action via these receptors probably contributes to the anti-nociceptive actions of somatostatin administered intrathecally or systemically [18], [85].

Since sst_2A_ is restricted to around half of the inhibitory interneurons in the superficial dorsal horn, and is differentially expressed by specific neurochemical types [20], this should allow the roles of these cells to be investigated by using intrathecal injections of saporin conjugated to somatostatin or its analogues to ablate these cells selectively [86]. Studies with other peptide conjugates of saporin have shown that while these selectively destroy populations of spinal neurons, they do not appear to damage primary afferents that express the corresponding receptors [87]–[89].

### NK1r expression in the mouse dorsal horn

Using the same NK1r antibody and a similar counting method, we estimated that in laminae I, II and III of the rat, the proportions of neurons that expressed the receptor were 45%, 6% and 11%, respectively [16]. Although the result obtained for lamina III is somewhat lower in the mouse, the most striking difference is in lamina I, where NK1r-immunoreactive neurons account for less than 20% of all neurons in the mouse (i.e. less than half of the proportion seen in the rat). Lamina I of the rat spinal cord has been shown to contain two different types of NK1r^+^ cell that differ in size and receptor expression level: projection neurons and interneurons [90], [91]. The projection neurons, virtually all of which belong to the spinoparabrachial tract [2], have relatively large cell bodies and usually show strong NK1r immunoreactivity. In contrast, the interneurons have significantly smaller cell bodies and invariably show weak or moderate NK1r immunostaining. Since virtually all NK1r^+^ cells in the superficial laminae lack GABA immunoreactivity [29], these interneurons are thought to be excitatory. Projection neurons are highly concentrated in lamina I in the rat, but only make up around 5% of the neuronal population, and ∼80% of them are NK1r-immunoreactive [92]. We therefore estimate that 90% of the NK1r^+^ cells in lamina I of the rat are excitatory interneurons, and that more than half of the excitatory interneurons in this lamina possess the receptor.

There have apparently been few anatomical studies of lamina I projection neurons in the mouse, but we have found that as in the rat, many of these cells are strongly NK1r-immunoreactive (AJ Todd, unpublished observations). Wang et al. [70] recently reported that there were 100 retrogradely labelled lamina I cells per twenty 25 µm thick transverse sections from lumbar spinal cord in mice that had received large Fluorogold injections into the lateral parabrachial area on one side. Based on the length of the L4 segment in our study, this would equate to ∼290 cells in the L4 segment. However, they included both contralateral and ipsilateral cells, and we have shown that in the rat, most ipsilateral spinoparabrachial cells project bilaterally, and that these account for 30% of all lamina I spinoparabrachial neurons [92]. If a similar arrangement is present in the mouse, then the number of lamina I spinoparabrachial cells on one side would be approximately 220, and since we estimate that there are ∼4500 neurons in lamina I in this segment, this would correspond to 5% of the neurons in this lamina, a similar proportion to that estimated in the rat.

It is therefore likely that as in the rat, most of the NK1r-immunoreactive cells in lamina I are interneurons, although the proportion of these cells with the receptor is substantially lower than in the rat.
